# Design of optimal concentrations for in vitro cytotoxicity experiments

**DOI:** 10.1007/s00204-024-03893-1

**Published:** 2024-11-16

**Authors:** Leonie Schürmeyer, Chen Peng, Wiebke Albrecht, Tim Brecklinghaus, Pauline Baur, Jan G. Hengstler, Kirsten Schorning

**Affiliations:** 1 Department of Statistics, TU Dortmund University, Vogelpothsweg 87, Dortmund, 44227 North Rhine-Westphalia Germany; 2 Leibniz Research Centre for Working Environment and Human Factors at the Technical University of Dortmund (IfADo), Ardeystraße 67, Dortmund, 44139 North Rhine-Westphalia Germany; 3 Department of Microbiology, School of Medicine, Jiangsu University, Zhenjiang, 212013 China

**Keywords:** Optimal design, *D*-optimal design, Bayesian optimal design, Concentration-response experiments

## Abstract

Concentration-dependent cytotoxicity experiments are frequently used in toxicology. Although it has been reported that an adequate choice of concentrations improves the quality of the statistical inference substantially, a recent literature review of three major toxicological journals has shown that the corresponding methods are rarely used in toxicological practice. In this study the performance of different sets of concentrations, also called designs, are analyzed, while the overall goal is to promote the advantages of optimal design procedures and to present a user-friendly guideline for planning new cytotoxicity concentration-response experiments. We compare the frequently used log-equidistant design to a Bayesian design, which is constructed by methods of optimum design theory. Using both a dense data set of concentration-cytotoxicity data of valproic acid (VPA) and regular assay data of 104 substances, the performance of the different designs is analyzed in two scenarios, where detailed previous knowledge on VPA is available or not. The results show that it is critical to apply a specific design strategy to determine optimal concentrations for cytotoxicity testing. In particular, the Bayesian design technique with and without incorporating pre-existing knowledge of a specific test substance resulted in a more precise statistical inference than the other used designs. Finally, we present a guideline for upcoming experiments and an accessible user-friendly Shiny app (see http://shiny.statistik.tu-dortmund.de:8080/app/occe).

**Supplementary Information:**

The online version contains supplementary material available at 10.1007/s00204-024-03893-1.

## Introduction

Concentration-dependent cytotoxicity tests are frequently used in toxicology (Vorrink et al. 2018; Brecklinghaus et al. 2022a; Proctor et al 2017; Khetani et al. 2013; Gu et al. 2018). These tests usually serve to determine the ${\text {EC}}_{50}$-value as the concentration of a substance that reduces vitality to 50% of solvent controls. In further studies, also the lowest concentrations that begin to cause cytotoxicity are analyzed by determining ${\text {EC}}_{10}$-values (Brecklinghaus et al. 2022b; Ghallab et al 2022). An important challenge of in vitro cytotoxicity testing is the choice of adequate test concentrations. Here, two scenarios should be differentiated, depending on the availability of previous established ${\text {EC}}_{10}$- and ${\text {EC}}_{50}$-values.

If ${\text {EC}}_{10}$- and ${\text {EC}}_{50}$-values are known from previous studies this can be used to plan the cytotoxicity experiment as a one-step procedure. We compare three procedures to select sets of experimental concentrations, also called designs, when prior knowledge on the ${\text {EC}}_{10}$ and ${\text {EC}}_{50}$ is available. First, the log-equidistant design technique can be applied, where the highest solubility or maximal concentration is multiplied or divided by the same factor. Second, the log-equidistant design technique can be combined with biological arguments so that, e.g., around the ${\text {EC}}_{50}$-value smaller distances of concentrations are chosen. Third, the pre-existing knowledge about the ${\text {EC}}_{10}$- and ${\text {EC}}_{50}$-value can serve as a basis for a determination of optimal test concentrations using a (pseudo) Bayesian design technique (see Chaloner and Larntz 1989). This technique has been developed within the broad research field of optimal design of experiments (ODE), which provides methods on planning and conducting experiments in a way that maximizes the efficiency of the data-generating process. Hereby, the major goal is to obtain the most informative and precise statistical results with the smallest necessary resources. In the context of toxicological experiments, this means identifying the smallest necessary number of different concentrations, their allocations and the necessary number of replicates.

Despite these advantages, the techniques provided by ODE, in particular the Bayesian technique, are rarely used for planning toxicological experiments. Instead, the log-equidistant technique is frequently applied to obtain sets of test concentrations in practice (see Kappenberg et al. 2023). This is also due to the fact, that the influence of the test concentrations on the quality of data and thus on the quality of the statistical analysis is usually unknown. As a result, the design of test concentrations is often neglected during the planning of an experiment (see again Kappenberg et al. 2023). Besides, the limited availability of online tools for the calculation of optimal test concentrations based on Bayesian or other ODE techniques makes its application difficult for practitioners (Holland-Letz and Kopp-Schneider 2021).

To compare the performance of the three different design techniques in concentration-cytotoxicity experiments, reference cytotoxicity data for a single test substance was produced with an unusually high number of concentrations and biological replicates. The test substance under consideration was valproic acid (VPA). VPA is a medium soluble compound with well-documented human hepatotoxicity and high clinical relevance. Besides it has been used as a positive control in previous studies (Albrecht et al. 2019). The curve fitted to this atypically large data set is assumed to represent the true concentration-cytotoxicity relationship of VPA, hereafter referred to as reference curve. Then the quality of each considered design was assessed by comparing it against the reference curve, thereby using only a subset of the full VPA data set that corresponds to the design.

An additional research question of this study was the analysis of a sequential approach with a pre-experiment followed by a main experiment. Here, the data from the pre-experiment serve as prior information for the determination of the design that should be used in the main experiment. Each of the considered design techniques was used to plan the pre-experiment. Based on the corresponding subsets of the large data set, it was investigated, if the experiment could be improved by the sequential approach. Moreover, it was analyzed which of the design techniques led to the best results if used as a design in the pre-experiment.

So far, the proposed design techniques all require pre-existing knowledge about the properties of the considered test substance. However, this information may not be available in some situations, for example when a new substance is tested. To address such research situations, a second scenario was considered where available data of other, related test compounds were used as less specific prior knowledge. More precisely, already existing concentration-dependent cytotoxicity data of 104 other compounds that had been tested by the same cytotoxicity assay were used to construct (less specific) prior information about the test substance. This prior information was used to apply the different design techniques. In particular, a Bayesian design was constructed that could be used both for experiments on the test substance VPA itself and for experiments on all other considered substances.

Finally, we present a guideline with easy-to-apply methods for one-step and sequential strategies of toxicity testing. Furthermore, we provide a Shiny app for a user-friendly calculation of optimal concentrations for upcoming experiments.

## Materials and methods

### Test compounds

For this study a compound set with a total of 104 compounds, composed mostly of pharmaceuticals, was utilized. Details on the compounds, supplier, utilized solvents and concentrations are provided in Supplement Data.

### Cytotoxicity testing in HepG2 cells

The HepG2 cell line (ATCC number: HB-8065™) was used for the cytotoxicity tests as described previously (Brecklinghaus et al. 2022a; Albrecht et al. 2019). Briefly, the cells were cultured in 4.5 g/L glucose Dulbecco’s modified eagle’s medium (PAN Biotech GmbH, Aidenbach, Germany P04-04500) supplemented with 10% heat inactivated fetal calf serum (PAN Biotech GmbH, Aidenbach, Germany 3702-P103009) and 100 U/ml Penicillin/0.1 mg/ml Streptomycin (PAN Biotech GmbH, Aidenbach, Germany P06-07100). Approximately $15\,000$ cells were seeded in black 96-well plates (Greiner bio-one, Frickenhausen, Germany, REF 655986) coated with 0.25 mg/ml rat tail collagen (Roche Diagnostic Mannheim, 10 mg, Cat. No. 11171179001). No cells were seeded into the outermost columns and rows. These wells were filled with phosphate buffered saline to counteract evaporation effects. After 16–20 h incubation at 37 $^{\circ }\hbox {C}$ and 5% $\text {CO}_2$ the medium was exchanged and exposure to the test compounds was started. For each test compound a vehicle control was included. The cells were exposed to the test compounds for 48 h at 37 $^{\circ }\hbox {C}$ and 5% $\text {CO}_2$. At the end of the exposure period, the medium was removed, the cells were washed three times with phosphate buffered saline and 100 $\mu$l/well of 1:5 CellTiter-Blue (short: CTB) reagent (Promega, Cat. No. G8081) in medium were added. As a background control wells with CTB mixture without cells was included. The cells were further incubated at 37 $^{\circ }\hbox {C}$ and 5% $\text {CO}_2$ until a color change from blue to purple was observed in the vehicle controls. The fluorescence was measured with the Tecan Infinite M200 Pro plate reader (software i-control, version 1.7.1.12) utilizing the excitation wavelength 540 nm and emission wavelength 594 nm. Prior to curve fitting the mean of the background control wells was subtracted from all samples. Each compound was tested in at least three biological replicates with at least three technical replicates. The raw data for all compounds are provided in Supplement Data. In the following analysis the dense data set of VPA is referred to as VPA data set, besides the collection of data of 104 compounds investigating Drug-induced liver injury (short: DILI) effects is referred to as DILI data set. A part of the DILI data set was already published before (Albrecht et al. 2019). Since the blue resazurin contained in the CellTiter-Blue reagent is metabolized by vital cells to the pink resorufin, the concentration dependent change in the measured fluorescence corresponds to a change in viability of the cells.

### Statistical methods

All analyses were performed using the statistical software R, version 4.2.2 (R Core Team 2022) and Julia, version 1.9.3 (Bezanson et al. 2017), especially the Julia package Kirstine.jl (Sandig 2024).

#### Modelling cytotoxic concentration-response data

The concentration-response relationships of the substances investigated within the paper are expected to follow a sigmoidal course, so that the corresponding concentration-response data is always fitted to a four-parametric log-logistic model, hereinafter also referred to as 4pLL-model. Following Ritz et al. (2015), a 4pLL-model consists of four parameters and is defined by the non-linear regression function

1 $$\begin{aligned} \eta (x, \theta ) = c + \frac{d-c}{1+\exp \left( b(\log (x)-\log (e))\right) }, \quad \theta =\left( b,c,d,e\right) , \end{aligned}$$

where *x* is a concentration within the concentration range ${\mathcal {X}}=[0, x_{\max } ]$. The lower and upper asymptotes are represented by the parameters *c* and *d*, whereas the parameter *b* describes the steepness of the curve. Furthermore, the parameter *e* describes the turning point of the curve, i.e. the concentration on the S-shaped curve halfway between *c* and *d*.

Let now $\{x_1, \ldots , x_K\}$ be a set of selected concentrations, where $x_k \in {\mathcal {X}}=[0, x_{\max }]$ and $K \in {\mathbb {N}}$. This set will be denoted by design in the following. We assume that *n* replicates $y_{k1}, \ldots , y_{kn}$ are produced at each concentration $x_k$ of the design during the experiment such that the resulting concentration-response dataset is given by $\{(x_k, y_{kj})|k=1, \ldots , K, j=1, \ldots , n\}$.

For the fit of a 4pLL model to the dataset, least squares (LS) estimation is used. More precisely, the LS estimate ${\hat{\theta }}$ is defined as the parameter value that minimizes the quadratic distance between the data and the function in (1), i.e.

$$\begin{aligned} {\hat{\theta }} \text{ minimizes } \sum _{k=1}^K \sum _{j=1}^{n} (y_{kj}-\eta (x_k, \theta ))^2\,. \end{aligned}$$

The fitted 4pLL function is then given by $\eta (x, {\hat{\theta }})$. To measure the precision of the fitted 4pLL function, the covariance matrix of the LS-estimate ${\hat{\theta }}$, denoted by $\text{ Cov }({\hat{\theta }})$, is considered. As the direct calculation of this covariance matrix is difficult due to the non-linearity of the 4pLL function, an approximation is used. Following Jennrich (1969), the covariance matrix can be approximated by the inverse of the information matrix $M(x_1, \ldots , x_K, \theta )$ which is given by

2 $$\begin{aligned} M(x_1, \ldots , x_K, \theta ) = \sum _{k=1}^K \frac{\partial }{\partial \theta }\eta (x_k, \theta )\frac{\partial }{\partial \theta }\eta (x_k, \theta )^\top \,. \end{aligned}$$

Here, $\frac{\partial }{\partial \theta }\eta (x_k, \theta )$ denotes the gradient of the regression function, i.e. of the function given in (1), and $\theta$ is the unknown true parameter of the model. Consequently, the precision of the LS estimate ${\hat{\theta }}$ (and thus the precision of the model fit) depends both on the unknown parameter $\theta$ and the concentrations involved in the design. Note that the variance does not depend directly on the responses, but on the concrete model assumption made in advance. Therefore, no observations are needed to determine concentrations that result in a precise estimator of $\theta$ if prior knowledge of the model parameter $\theta$ is available. For the sake of simplicity, we first assume that the unknown parameter $\theta$ is given by a fixed value $\theta _0\in {\mathbb {R}}^4$. Then, the target of optimal design theory is to determine the design $\{x_1,\ldots , x_K\}$, that results in the best precision of the LS estimate given that $\theta = \theta _0$. This optimization problem is equivalent to the maximization of the information matrix $M(x_1, \ldots , x_K, \theta _0)$ with respect to $x_1, \ldots , x_K$ and $K\in {\mathbb {N}}$. As matrices cannot be ordered, real-valued functions of the information matrix $M(x_1, \ldots , x_K, \theta _0)$ have to be incorporated to solve the optimization problem. Different real-valued functions can be used here, whereas the most popular real-valued function is the determinant (see for instance Pukelsheim 2006). More precisely, a design is called locally *D*(eterminant)-optimal if it maximizes

$$\begin{aligned} \log (\det (M(x_1, \ldots , x_K, \theta _0))) \end{aligned}$$

among all designs on the concentration range ${\mathcal {X}}=[0,x_{\max }]$ (see Chernoff 1953 among many others). The *D*-optimal design minimizes the volume of the confidence ellipsoid for the complete parameter $\theta$ (see again Pukelsheim 2006). Kiefer (1974) proved that the *D*-optimal design also minimizes the maximum (asymptotic) variance of the estimated regression curve within ${\mathcal {X}}$, i.e. the expression

$$\begin{aligned} \max _{x\in {\mathcal {X}}} \frac{\partial }{\partial \theta }\eta ^\top (x, \theta ) M^{-1}(x_1, \ldots , x_K, \theta )\frac{\partial }{\partial \theta }\eta (x, \theta )\,. \end{aligned}$$

Consequently, optimizing based on the *D*-optimality criterion results in a design that has a good overall performance.

If a real-valued function of the parameter $\theta$, i.e. $\mu (\theta )$, is of special interest, optimality criteria can be defined to measure the approximate variance of the corresponding estimator $\mu ({\hat{\theta }})$. More precisely, a design is then called locally *c*-optimal (with $c= \tfrac{\partial }{\partial \theta }\mu (\theta )$) if it minimizes its asymptotic variance

$$\begin{aligned} \tfrac{\partial }{\partial \theta }\mu ^\top (\theta )M^{-}(x_1, \ldots , x_K, \theta ) \tfrac{\partial }{\partial \theta }\mu ^\top (\theta ) \end{aligned}$$

among all designs on the concentration range ${\mathcal {X}}$. Here $M^{-}$ denotes the generalized inverse of *M*. Although using *c*-optimal designs results in the precise estimation of $\mu (\theta )$, an estimation of the complete parameter $\theta$ might not be possible if this design is used. For instance, the *c*-optimal design for estimating the effect at concentration $x_0$ consists of one concentration, namely $x_0$. While the observations at concentration $x_0$ can be used to estimate $\eta (x_0, \theta )$, they are insufficient to fit the complete regression curve $\eta (x, \theta )$ (Silvey 1980). One possibility to overcome this problem is to consider compound optimality criteria (McGree et al. 2008) combine different optimality criteria, such as the *D*-optimality criterion and a *c*-optimality criterion of interest. However, the compound criteria can become very complex and the *c*-optimality criterion has to be defined individually. Due to these pitfalls, we decided to use the *D*-optimality criterion for the further analysis.

Note that *D*-optimal designs depend on the choice of the parameter value $\theta _0$ and are therefore called **locally** *D*-optimal designs. When less specific prior knowledge about the unknown parameter $\theta$ is available, the concept of locally *D*-optimal designs can be extended to Bayesian *D*-optimal designs (Chaloner and Larntz 1989). Assume prior information about $\theta$ is available in the form of different potential values, e.g., $\theta _1, \ldots , \theta _P$. Then a design is called Bayesian *D*-optimal (for the prior $\theta _1, \ldots , \theta _P$) if it maximizes the function

$$\begin{aligned} \sum _{j=1}^P \frac{1}{P} \log (\det (M(x_1, \ldots , x_K, \theta _j))) \end{aligned}$$

with respect to $x_1, \ldots , x_K$ and $K\in {\mathbb {N}}$. Consequently, the resulting Bayesian *D*-optimal design maximizes the average of all potential determinants of the different possible information matrices. While we restrict ourselves to the case, where the potential parameter values obtain uniform weight (i.e. $\frac{{\text {1}}}{{\text {P}}}$), other weightings are possible using more complex prior distributions. During the study, the calculation of Bayesian *D*-optimal designs using a uniform prior on a given set of potential parameter values is called Bayesian design technique.

In cytotoxicity-testing, it is of interest at which concentrations a prespecified fraction of cells is damaged. Under the assumption that the concentration-response relationship is described by a 4pLL-model, these concentrations can be identified using the model fit of the data. More precisely, standardizing the upper asymptote *d* to 100%, as it is often done in cytotoxicity experiments (see Kappenberg et al. 2020), the concentration at which the fitted regression function $\eta (x, {\hat{\theta }})$ attains $50\%$ defines the value, at which exactly 50% of all cells were damaged. This value is denoted by the (absolute) ${\text {EC}}_{50}$-value. Note that the definition can easily be extended. For other percentages $p\%\in [0, 100]\%$, the (absolute) $\text {EC}_{p}$-value is defined by the concentration at which the fitted regression function $\eta (x, {\hat{\theta }})$ equals $p\%$, i.e. at which exactly $p\%$ of the cells were damaged.

## Results

### Design of an experiment with pre-existing knowledge about VPA

#### Construction of the concentrations for VPA data

Before a cytotoxicity experiment is conducted, the set of concentrations has to be fixed. For the determination of the sets of concentrations used to generate the dense dataset of the test substance valproic acid (VPA) (see Supplement Data) three different design techniques were applied.

The here applied design techniques require pre-existing knowledge about the test substance VPA, which was provided in form of data from three former experiments. More precisely, the highest possible soluble concentration was identified by 46.4 mM, resulting in the concentration range ${\mathcal {X}} = \left[ 0,46.4\right]$ mM. Moreover, potential regions of ${\text {EC}}_{10}$- and ${\text {EC}}_{50}$-values were given by [0.373, 0.756] mM and [6.0, 7.0] mM, respectively. Finally, it was assumed that the effect at the solvent control is equal to 100, whereas the response/effect converges to zero if the concentration tends to infinity.

**Fig. 1:**
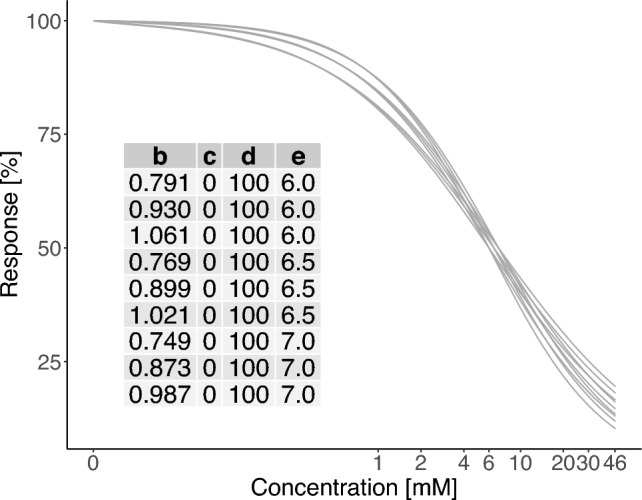
Model curves of prior distribution based on ${\text {EC}}_{10}$- and ${\text {EC}}_{50}$-values of the pre-experiments of VPA with information of the corresponding parameters of the 4pLL-models. Using different model curves as prior information, we can ensure robustness

The three different design techniques were initially used to determine designs, which consist of four different concentrations. Applying the log-equidistant design technique, the design was constructed by a dilution factor of ten starting with the highest solubility 46.4 mM$\, \approx 10^{5/3}$ mM. This procedure resulted in the set of concentrations given by $\left\{ 0,0.464,4.64,46.4\right\}$ mM, which is called log-equidistant design in the following. Combining the log-equidistant technique with biological arguments led to the set of concentrations given by $\left\{ 0,1,10,46.4\right\}$ mM, which is called IfADo design in the following. Note that the IfADo design also includes a second concentration that is greater than 5 mM, which was chosen based on previous knowledge on the relatively high ${\text {EC}}_{50}$. Finally, the Bayesian design technique (cf. “Statistical methods”) was used. Hereby, it was assumed that the unknown concentration-response relationship of VPA could be described by a 4pLL-model. Additional necessary prior information about the parameters of the 4pLL-model was obtained by pre-existing knowledge: the asymptotes of the true concentration-response relationship were set to $c= 0$ and $d=100$ and three potential values each were provided for the ${\text {EC}}_{10}$- and ${\text {EC}}_{50}$-levels, respectively. The corresponding potential parameter values *b* and *e* were calculated for the $3 \times 3$ combinations of the ${\text {EC}}_{10}$- and ${\text {EC}}_{50}$-values resulting in a prior distribution of 9 potential model parameters. The corresponding curves are presented in Fig. 1. The resulting Bayesian *D*-optimal design, denoted by Bayesian design in the following, is given by the set of concentrations $\left\{ 0,1.229,9.334,46.4\right\}$ mM.

To compare designs with different numbers of concentrations, the initial designs (log-equidistant, IfADo and Bayesian) were extended progressively to sets of five, six, and seven concentrations (see Table 1). Hereby, the log-equidistant design was first extended by a dilution factor of ten, which resulted in adding the concentration 0.0464 mM. As a progressive dilution of ten for more than five concentrations was inappropriate, the log-equidistant design was extended using a dilution factor of $10^{1/3}$ for 4.64 mM and then for 46.4 mM instead, resulting in the concentrations $(10)^{1/3}=2.154$ mM and then $(10)^{4/3}=21.54$ mM. The IfADo design was extended by a dilution factor of ten to designs with five and six concentrations, respectively. Finally, the concentration $(10)^{4/3}=21.54$ mM was also added to the IfADo design. The Bayesian design was extended to reduce the absolute distances between the different concentrations. Therefore, the gaps between the present concentrations of the Bayesian design were filled up with additional concentrations from largest to lowest determined by the available concentration closest to the midpoints of the present concentrations, in particular with 21.54 mM, 5.432 mM, and finally with 0.486 mM.

**Table 1:** Designs under consideration with concentrations (in mM) included in the VPA data set

Design	$\sharp$Conc	Concentrations						
IfADo	4	0			1	10		46.4
IfADo	5	0		0.1	1	10		46.4
IfADo	6	0	0.01	0.1	1	10		46.4
IfADo	7	0	0.01	0.1	1	10	21.54	46.4
Log-equidistant	4	0		0.464		4.64		46.4
Log-equidistant	5	0	0.0464	0.464		4.64		46.4
Log-equidistant	6	0	0.0464	0.464	2.154	4.64		46.4
Log-equidistant	7	0	0.0464	0.464	2.154	4.64	21.54	46.4
Bayesian	4	0		1.229		9.334		46.4
Bayesian	5	0		1.229		9.334	21.54	46.4
Bayesian	6	0		1.229	5.432	9.334	21.54	46.4
Bayesian	7	0	0.486	1.229	5.432	9.334	21.54	46.4

The log-equidistant, the IfADo, and the Bayesian designs were incorporated in the new experiments planned with sets of 50 concentrations. Those 50 concentrations further comprise the concentrations of locally *D*-optimal designs corresponding to the parameter combinations displayed in Fig. 1 (see Chernoff 1953 for details) and concentrations incorporated due to biological arguments. The data set, especially the values of the 50 different concentrations can be found in the Supplement (see Supplement Data).

#### Definition of a reference curve for VPA data

In routine toxicity testing, concentrations are often defined by a log-equidistant design, which means that the concentrations are multiplied by the same dilution factor. In a first step, we studied whether the Bayesian design or the log-equidistant design leads to better model precision.

**Fig. 2:**
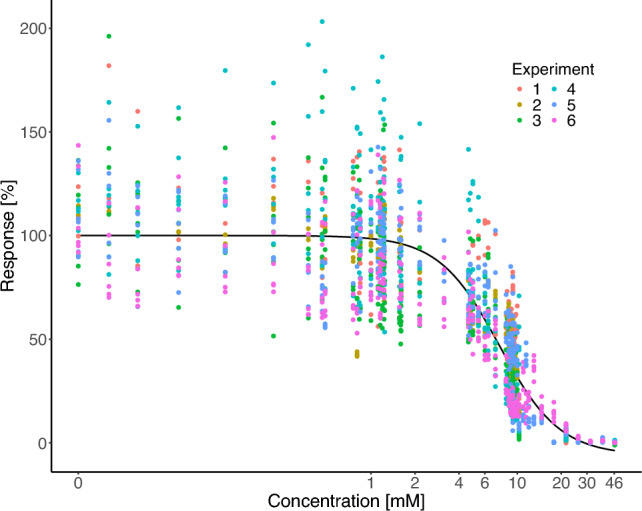
Cytotoxicity values for the six independent experiments of the VPA data set, 4pLL reference curve presented in black

A precondition for the comparison of the precision of the different design techniques is a reference curve. The reference curve was obtained from cytotoxicity analyses of the test compound valproic acid (VPA) in six independent experiments with 50 concentrations each and all data points were fitted together to a 4pLL-model (Fig. 2). The purpose of using many concentrations and experiments was to approximate the true concentration–cytotoxicity relationship that theoretically would be approached when the number of experiments and concentrations grows to infinity.

#### Bayesian design leads to a higher model precision compared to a log-equidistant design

In the following, the different presented design techniques to plan a cytotoxicity experiment are compared. First, the Bayesian design technique is compared to the frequently used log-equidistant design. To study the performance of both methods in the context of the different number of used concentrations, we consider designs with 4, 5, 6, or 7 concentrations based on the two design techniques, whereby the solvent control (where the VPA concentration was zero) is also counted as concentration (see Table 1). Note that for both methods, the highest soluble concentration (46.4 mM) was used as a starting point for dilutions.

**Fig. 3:**
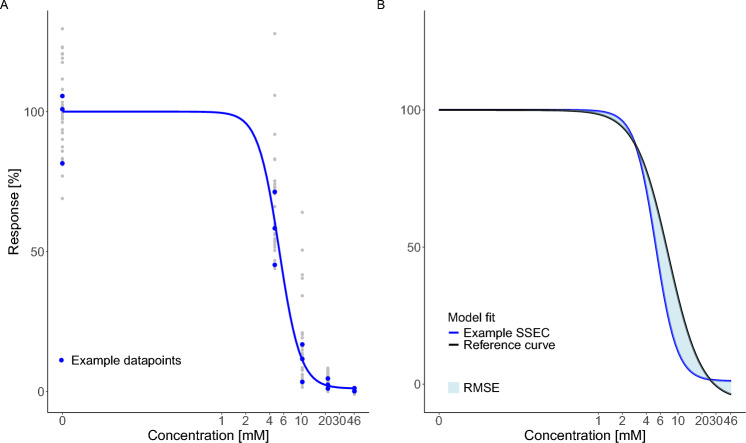
**A** Example illustrating the construction of a SSEC using the Bayesian design with five concentrations. The gray points depict all possible cytotoxicity values within the VPA dataset, while the blue data points represent three randomly selected cytotoxicity values, each representing a technical replicate. **B** Visualization of the RMSE of an exemplary SSEC compared to the reference curve. The greater the area between the two curves is, the higher is the RMSE, indicating a greater deviation from the reference curve

**Fig. 4:**
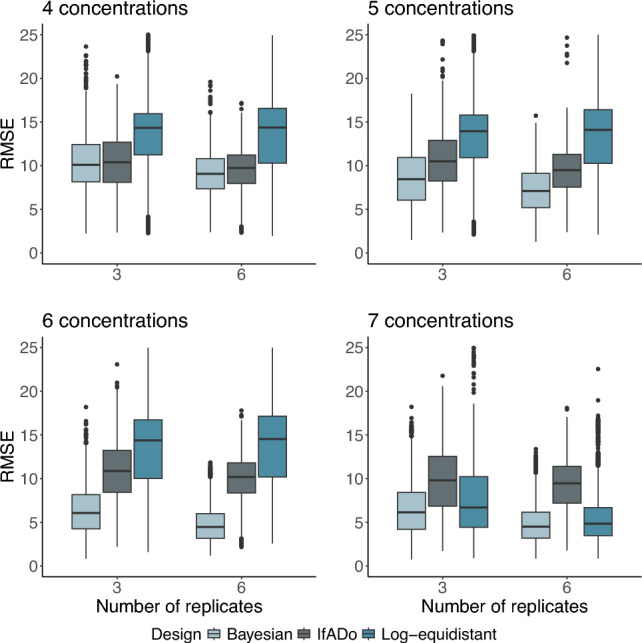
RMSE-values grouped by design, number of concentrations and used replicates for SSECs. Small RMSE values indicate higher precision of the design

To simulate specific scenarios that could represent individual experiments, three or six of the 30 observations (cytotoxicity) were randomly chosen for each selected concentration. With these specific scenarios, real concentration-response experiments were mimicked using actual data points, allowing for realistic variances and errors at different concentration levels to be accurately reflected. For each scenario, a 4pLL-model was fitted to the corresponding concentration-cytotoxicity data, further named ‘scenario specific estimated curve’ (SSEC). An example of the construction of a SSEC is shown in Fig. 3A. This simulation procedure was performed 3000 times for each design. To assess model precision in a practical manner, each SSEC was compared to the reference curve by calculating the Root Mean Squared Error (RMSE). The RMSE, represented by the area between the two curves (Fig. 3B), was used to make the differences easier to interpret. Additionally, we analyzed the maximal distance of the SSECs from the reference curve, as the *D*-optimality criterion is connected to the *G*-optimality criterion that seeks to minimize the maximal asymptotic variance (see “Modelling cytotoxic concentration-response data”). The smaller the RMSE/maximal distance, the higher the similarity of an SSEC to the reference curve. The resulting 3000 RMSEs for each design are summarized by box plots (Fig. 4), whereas the corresponding box plots of the maximal distances are depicted in Figure S1 in the Supplement Figures.

The results demonstrate consistently lower RMSEs (better) for the Bayesian design compared to the log-equidistant design strategy (Fig. 4). This is obtained for 4, 5, 6 as well as 7 concentrations and is consistent if three or six replicates were chosen. However, the difference in RMSEs between the Bayesian and the log-equidistant design strategy decreases if 7 concentrations are chosen, compared to the scenarios with 4–6 concentrations. This difference is explained by the fact that the critical concentration of 21.54 mM becomes involved in both designs only when 7 concentrations are applied. Since the Bayesian design already includes higher concentrations when 4, 5, and 6 concentrations are chosen (explained in more detail in the discussion, see “Discussion and conclusion”), the improvement of model precision was higher for the log-equidistant design. Considering the maximum distance (see Figure S1 in Supplement Figures) similar results can be observed.

In laboratory routine, concentrations for cytotoxicity testing are often chosen based on previous experience or published data of a test substance, whereby smaller distances of concentrations are chosen in the concentration range of the expected ${\text {EC}}_{50}$. Usually, the choice of these concentrations is not based on a mathematical procedure but on previous knowledge, for example, former experiments showing in which concentration range a substance begins to be cytotoxic. Based on the experience of former experiments (see Albrecht et al. 2019), the concentrations of the so-called “IfADo design” were chosen in laboratory routine and also listed in Table 1.

The IfADo design technique is outperformed by the Bayesian design technique in terms of RMSE. While for 4 concentrations the mean RMSE of the IfADo design is only slightly higher (worse) than the RMSE of the Bayesian design, the latter is superior if 5, 6, or 7 concentrations are used (Fig. 4).

**Fig. 5:**
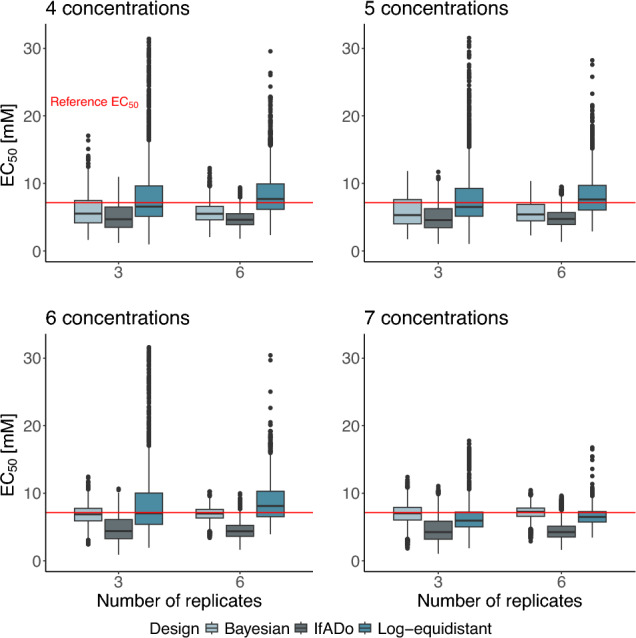
${\text {EC}}_{50}$-values grouped by design, number of concentrations and used replicates for SSECs. The red line corresponds to the reference ${\text {EC}}_{50}$. The closer the ${\text {EC}}_{50}$-values of the SSECs are to the reference value, the more precisely the ${\text {EC}}_{50}$s are estimated

Moreover, the influence of the different design techniques on the precision of the ${\text {EC}}_{50}$-value (defined as the concentration that reduces vitality to 50% of the solvent control) was analyzed using the scenario-specific simulation. More precisely, we calculated the ${\text {EC}}_{50}$-values of the 3000 SSECs for each design and compared them with 7.147 mM, which is the ${\text {EC}}_{50}$-value of the reference curve. The results are presented in Fig. 5, where the red lines represent the ${\text {EC}}_{50}$-value of the reference curve. For the interpretation of the results, it is important to consider both the median and the interquantile ranges (IQR) of the ${\text {EC}}_{50}$-values.

In general, the Bayesian design approach leads to the most precise ${\text {EC}}_{50}$-estimations compared to the other design approaches although the Bayesian design is not constructed for that purpose. Considering the cases in which the number of different concentrations is 6 or 7, the Bayesian ${\text {EC}}_{50}$ median is closer to the reference than the one of the log-equidistant design. For 4 and 5 concentrations, the median ${\text {EC}}_{50}$ obtained by the Bayesian design shows a higher deviation from the reference than the ${\text {EC}}_{50}$ median obtained using the concentrations of the log-equidistant design (Fig. 5). Besides, the IQRs of the ${\text {EC}}_{50}$-values based on the Bayesian design technique are smaller than the ones based on the log-equidistant design technique in the situation of fewer than 7 concentrations. Comparing the designs consisting of 7 concentrations, the IQRs are of a similar size.

The precision of the ${\text {EC}}_{50}$ based on the IfADo design is worse than the one of the Bayesian and the log-equidistant design techniques. In particular, the IfADo design leads to a substantial deviation of the median ${\text {EC}}_{50}$ to the reference independent of the number of different concentrations involved.

The number of outliers produced, if the different design techniques are used, is also an indicator of the quality of the experiment. Considering both the RMSE-values (see Fig. 4) and the ${\text {EC}}_{50}$-values (see Fig. 5), the outliers for the Bayesian design technique are lower than for the log-equidistant design technique, independent of the number of concentrations used. Therefore, the Bayesian design leads to RMSEs and ${\text {EC}}_{50}$-values with substantially lower variability.

In toxicological experiments sometimes a sequential design of an experiment consisting of a pre-experiment and a main experiment is necessary. Therefore, a sequential design approach, involving a preliminary experiment followed by a main experiment, was evaluated to determine whether it could enhance the precision of concentration-dependent toxicity testing. The preliminary experiment is used to obtain knowledge about the ${\text {EC}}_{10}$-value and the ${\text {EC}}_{50}$-value of the test substance. This information is then utilized to refine the design of the main experiment, with the goal of obtaining more detailed insights into the cytotoxicity of the substance. It was investigated if the sequential procedure improves the performance of the different considered design techniques. The performance of the log-equidistant design and the IfADo design is improved by the sequential approach, whereas the performance of the Bayesian design is not affected. However, the sequential IfADo approach remains worse than the non-sequential Bayesian approach. The results indicate that a sequential experiment is not required when concentrations are initially selected using the Bayesian technique. For a comprehensive description of the analysis setup and detailed results, refer to the Supplement Sequential analysis.

#### Bayesian design requires the smallest sample size

An important aspect of planning a cytotoxicity experiment is the choice of a sample size that ensures a pre-specified statistical quality of the experiment. An appropriate quality indicator is the length of the confidence interval (LCI) of the estimate of the ${\text {EC}}_{50}$-value. The LCI decreases if the sample size increases, while its value also depends on the variance of the data and the used design, i.e. the set of considered concentrations. Therefore, using the variance of the VPA data set (see Fig. 2), the dependence between the required sample size and the resulting LCI was investigated for all designs displayed in Table 1.

**Fig. 6:**
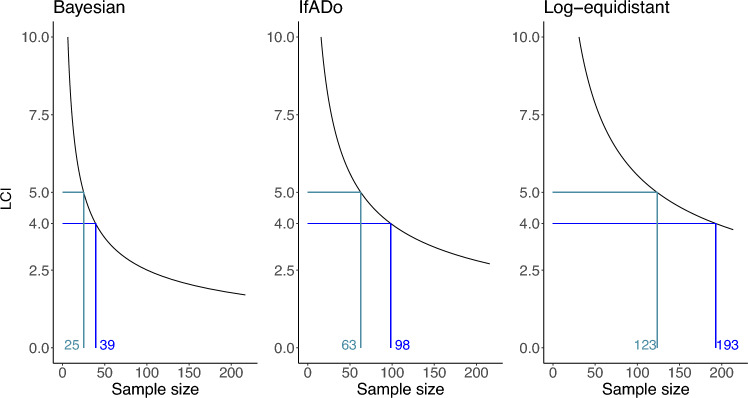
The relation between the sample size and the length of the confidence interval (LCI) of the ${\text {EC}}_{50}$-value is shown grouped by design. The blue lines indicate the number of observations required to achieve a specific level of precision for the ${\text {EC}}_{50}$ (LCI). To achieve the same precision of the ${\text {EC}}_{50}$ the Bayesian design requires considerably less observations than the other two design techniques

The Bayesian design technique requires a substantially smaller sample size than the other design techniques to achieve the same precision in terms of LCI (see Fig. 6 and Suppl. Figure S2). In Fig. 6, the whole relation between the sample size and the LCIs is visualized for the three different designs with seven concentrations, while the corresponding visualizations for the designs with smaller numbers of different concentrations can be found in Supplement Figure S2. The performance of the different designs can be compared by fixing a value for the LCI. For instance, if an LCI of 5 should be achieved and the Bayesian design with seven concentrations is used, a sample size of 25 is sufficient, while a five times larger sample size of 123 is necessary to achieve an LCI of 5 using the seven concentrations of the log-equidistant design. The corresponding required sample size of the IfADo design with seven concentrations is 63.

The LCI depends on the reference curve and the different concentrations in each considered design. This implies that increasing the number of concentrations does not necessarily decrease the LCI when the concentrations have already been optimally selected for a smaller set of concentrations (see Suppl. Figure S2).

Summarizing, the Bayesian design technique leads to the same precision with less experimental effort compared to the two other design techniques.

### Design of an experiment without prior knowledge of a test compound

Often, an cytotoxicity experiment has to be planned for a test substance for which no direct prior information is available. In this situation, less precise information about the behavior of related test compounds can be used, e.g., based on historical data. The case in which no information about the new substance can be gained rarely occurs. Furthermore, the design of an experiment without any prior information involves different statistical methods, which are not included in this study for the sake of brevity. Thus, this study focuses on the optimal design of a cytotoxicity experiment of a new (unknown) test substance where information about related test compounds is available.

#### Construction of a universal Bayesian design based on DILI data

Often, the cytotoxicity of a test substance has to be investigated without direct prior knowledge that can be used to design the experiment. In this situation, a sequential procedure that consists of a pre- and a main experiment is inevitable. Then, an important question is how the pre-experiment should be designed without direct prior information.

We investigated if the concentrations of the pre-experiment should be defined by a log-equidistant design, which is frequently used in routine cytotoxicity testing, or if the concentrations should be obtained by the Bayesian design technique using less informative prior knowledge. One option to generate this prior information is using available information about compounds that are assumed to be similar to the test compound under consideration.

**Fig. 7:**
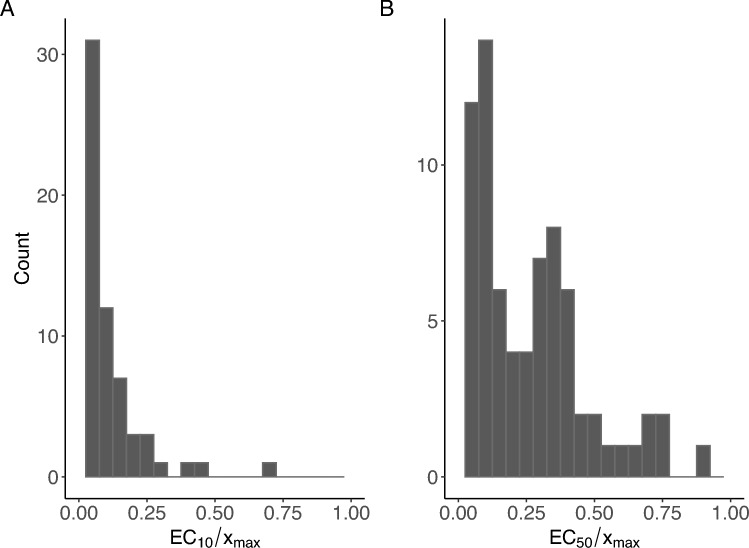
**A** Histogram of all ${\text {EC}}_{10}$-values of the considered substances in relation to the maximal concentration $x_{\max }$, **B** Histogram of all ${\text {EC}}_{50}$-values of the considered substances in relation to the corresponding highest considered concentration $x_{\max }$

For the substance under investigation, VPA, data of 103 other substances within a DILI data set (see Supplement Data) were used as (less informative) prior knowledge to construct a Bayesian design with seven concentrations. For each tested substance, determining the highest considered concentration depends on various limiting factors. The highest concentration is influenced by the highest solubility, as well as the pH value, and the requirement to include at least one concentration with no observable effect. As the concentration-response data of the different substances within the DILI data set vary substantially in the highest considered concentrations, the substance-specific data had to be normalized by dividing the set of originally used concentrations by the respective corresponding highest concentration. This normalization resulted in the same concentration range of all 103 substances, namely ${\mathcal {X}}_0=[0,1]$. The resulting data set is called the normalized DILI data set in the following. For each of the 103 substances within the normalized DILI data set the corresponding concentration-response data were fitted to a 4pLL-model. For 77 compounds the resulting model fits showed a goodness-of-fit value above 0.55 (see Albrecht et al. 2019), thus a convenient 4pLL-model fit was assumed here. The data of the remaining 26 substances were excluded from the further analysis, as they were inappropriate for the construction of suitable prior knowledge. Considering the distribution of the (normalized) ${\text {EC}}_{10}$ and ${\text {EC}}_{50}$, a major part of substances resulted in small values, respectively (Fig. 7). Only one substance, Troglitazone, had a normalized ${\text {EC}}_{50}$-value greater than 1 corresponding to an original ${\text {EC}}_{50}$ value greater than its highest considered concentration used in the experiment. Therefore, Troglitazone was excluded from further analysis. Summarizing, parameter estimations of the 4pLL-models fitted to 76 substances of the normalized DILI set served as prior distribution for the Bayesian design technique, whereby the corresponding 76 parameter combinations were equally weighted. The concentration range for the Bayesian design technique was also standardized to the concentration range ${\mathcal {X}}_0=[0,1]$.

The number of concentrations was fixed to seven to develop a design that shows a high model precision and can easily be managed in a laboratory routine experiment. The resulting Bayesian design with prior information of the DILI data set (short: BayesianDILI) consisting of seven concentrations is then given by the set: $\{0, 0.0300, 0.0623, 0.1351, 0.3206, 0.5177, 1\}$. Note that the maximal concentration of this design is 1 and therefore neither directly applicable for the different substances within the original DILI set nor for VPA. To obtain an appropriate design for the substance under consideration the design has to be multiplied by the specified maximal concentration. For the example of VPA with a highest considered concentration of 46.4 mM, the BayesianDILI is then transformed to the design consisting of the concentrations 0, 1.392, 2.891, 6.269, 14.876, 24.021, 46.4 mM.

#### BayesianDILI design outperforms IfADo design for the example of VPA

If no prior knowledge about a test substance is available, an important question is how the corresponding experiment should be planned. The lack of prior information requires a sequential procedure consisting of a pre-experiment and a main experiment. The purpose of the pre-experiment is to gain some knowledge about the test substance, which then can be used for the design of the main experiment. The design of the pre-experiment itself is often chosen based on previous experience in laboratory routines. Besides a log-equidistant design technique is often applied, where the concentrations are chosen by a specified dilution factor. However, the application of a Bayesian design technique might be more suitable for the design of the pre-experiment.

In the following, it is investigated if a Bayesian technique based on indirect prior knowledge is superior to the currently established design strategies for planning a pre-experiment. More precisely, in the situation where the new substance is given by VPA, the BayesianDILI design given by the set $\{0, 1.392, 2.891, 6.269, 14.876, 24.021, 46.4\}$ mM (see previous Section) is compared to the log-equidistant, the IfADo and the Bayesian design with seven concentrations (see Table 1). For the comparison, simulations are performed based on the experimentally performed VPA dataset.

As this dataset does not include exactly the concentrations of the BayesianDILI design, the seven concentrations closest to the experimentally determined (“true”) concentrations were selected. The resulting set of seven concentrations is given by $\{0, 1.229, 2.154, 6.331, 10.287, 21.54, 46.4\}$ mM, which is called BayesianDILI_VPA design in the further analysis. Similar to previous analyses, the SSECs for the BayesianDILI_VPA design were calculated and compared to the reference curve in terms of RMSE and ${\text {EC}}_{50}$-values in each simulation step. The resulting values were then compared to the RMSEs and the ${\text {EC}}_{50}$-values of the SSECs of the log-equidistant, IfADo, and Bayesian design for VPA with seven concentrations (see Table 1). To prevent any ambiguity, the Bayesian design for VPA will be referred to as BayesianVPA in the following analysis. Note that the BayesianVPA design by construction has an advantage over the BayesianDILI_VPA design, because it is based on explicit prior knowledge about the substance VPA (see “Construction of the concentrations for VPA data”), whereas no knowledge about VPA itself was used to construct the BayesianDILI_VPA design (see “Construction of a universal Bayesian design based on DILI data”).

**Fig. 8:**
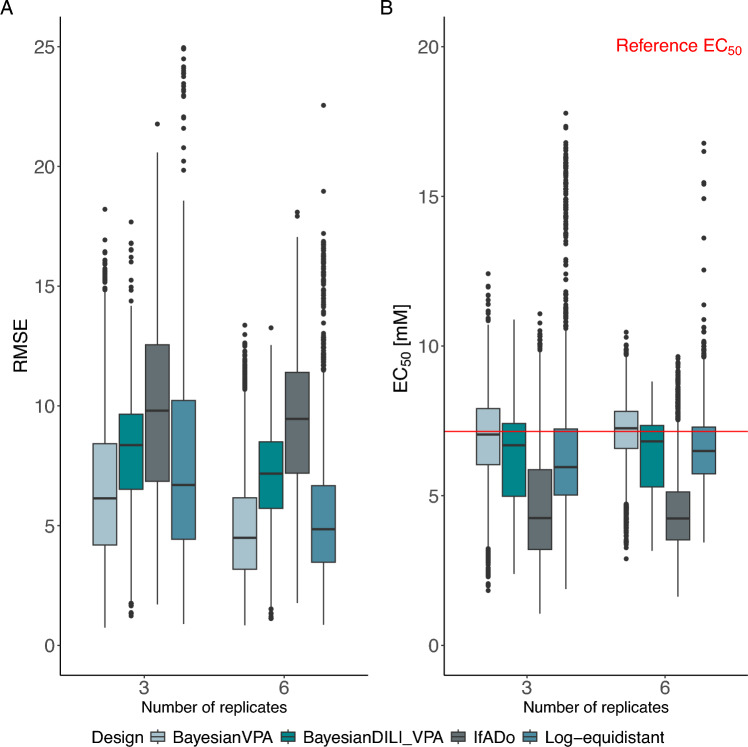
**A** RMSE-values grouped by design, number of concentrations and used replicates for SSECs. Small RMSE values indicate higher precision of the design. The BayesianDILI_VPA design performs better than the IfADo design although it does not incorporate prior knowledge about the test compound VPA itself. **B** ${\text {EC}}_{50}$-values grouped by design, number of concentrations and used replicates for SSECs. The red line corresponds to the reference ${\text {EC}}_{50}$. The closer the ${\text {EC}}_{50}$-values of the SSECs are to the reference value, the more precisely the ${\text {EC}}_{50}$-values are estimated. The BayesianDILI_VPA design outperforms the log-equidistant and IfADo design regarding the mean precision of the ${\text {EC}}_{50}$-values, although it does not incorporate prior knowledge about the test compound VPA itself

The results demonstrate a good performance of the BayesianDILI_VPA design compared to the three other design strategies, which incorporated direct prior knowledge about the new substance (Fig. 8A and B). This is obtained both for the RMSEs and the ${\text {EC}}_{50}$-values. In particular, the BayesianDILI_VPA design leads to RMSEs which lie between the RMSEs of the IfADo design and the ones of the BayesianVPA/log-equidistant design. Furthermore, the variability of the RMSE-values of the BayesianDILI_VPA design is lower than the variability of the other designs, indicated by smaller interquartile ranges. Concerning the ${\text {EC}}_{50}$, the BayesianDILI_VPA design results in a median estimate which lies below the ${\text {EC}}_{50}$ of the reference curve, but which is still better than the ones based on the IfADo design and the log-equidistant design, respectively (see Fig. 8B). Finally, the variability of the estimated ${\text {EC}}_{50}$ based on the BayesianDILI_VPA design is low compared to the log-equidistant design and only minor outliers are present.

#### BayesianDILI designs outperforms original and log-equidistant design for 76 substances of the DILI data set

In the following, the dependence of different design techniques on the quality of cytotoxicity experiments is investigated for substances of the DILI assay (except for VPA). More precisely, we considered the 76 substances of the DILI assay that served as prior information for the construction of the BayesianDILI design. Hereby, the BayesianDILI design was compared to the design originally used in the DILI assay for each substance (see Supplement Data) and to the frequently used log-equidistant design. For that purpose, the BayesianDILI design was rescaled based on the substance-specific highest considered concentration (see “ Construction of a universal Bayesian design based on DILI data” for details). Whereas the log-equidistant design with seven concentrations was constructed by using a dilution factor of 10 starting from the substance-specific highest considered concentration and is called log10 design in the following analysis. Since designs with different numbers of concentrations are not directly comparable, the sample size of all designs was fixed to the sample size of the original design using a rounding procedure (Pukelsheim and Rieder 1992).

For each substance, the performance of the different designs, i.e. the BayesianDILI, log10 and original design was analyzed using a simulation study. In contrast to the previous data-driven simulation, this analysis is based on computer-simulated data due to the limited availability of large datasets for the DILI substances. During the computer-based simulation, we assume normally distributed homoscedastic errors for all observations. This approach allows us to theoretically generate observations based solely on the model assumptions, eliminating the need for extensive experimental data. While this ensures greater comparability between simulations, it comes at the cost of losing the practical advantages of using real-world data, such as robustness and real-life applicability. However, the benefit of theoretical simulations lies in their flexibility and control over experimental conditions. This allows us to analyze the considered designs under idealized conditions and provides insights that might be challenging to obtain from empirical data due to variability issues. More precisely, this computer-based simulation is based on 1000 repetitions, which were conducted for each scenario, i.e. for each of the 76 substances using either the corresponding BayesianDILI, the original, or the log10 design. One simulation step consisted of the following intermediate steps: First, a 4pLL model was fitted to the original data of the considered substance and fixed as a reference curve for further analysis. Moreover, the corresponding substance-specific pooled variance was calculated based on the original data. Then new data was simulated using the responses of the reference curve at the concentrations of the considered design together with additive normally distributed random errors, whose variance coincided with the substance-specific pooled variance. Finally, a 4pLL model (SSEC) was fitted to the simulated data and compared to the substance-specific reference curve using the RMSE (see Fig. 3B for an illustration of this procedure).

**Fig. 9:**
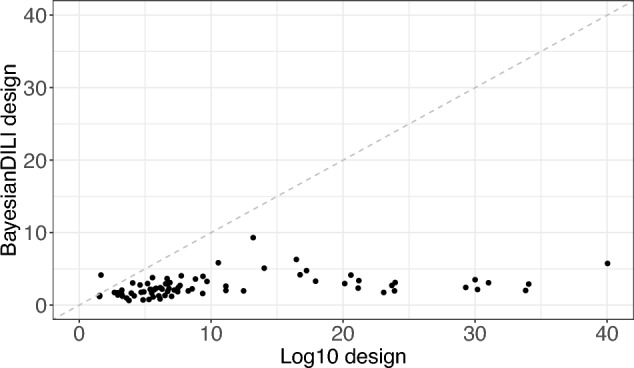
The Scatterplot shows RMSE-values corresponding to the simulation results of 76 substances regarding BayesianDILI and log10 design. Each dot represents one substance. The BayesianDILI design outperforms the log10 design for 75 of 76 substances

The resulting mean RMSE-values over the 1000 simulation steps of all 76 substances are shown for the BayesianDILI design on the *y*-axis versus the log10 design on the *x*-axis, where each dot represents one substance (Fig. 9). Values below the bisecting dashed line indicate a better performance of the BayesianDILI design compared to the log-equidistant one. Similarly, values above the dashed line show a better performance of the log10 design.

The simulation results show significantly lower mean RMSEs for the BayesianDILI design compared to the log10 design for 75 of 76 substances, with the exception of one compound (Griseofulvin) (Fig. 9). While the mean RMSEs based on the BayesianDILI design vary between 0 and 10, the values of the mean RMSEs based on the log10 design are up to four times higher.

**Fig. 10:**
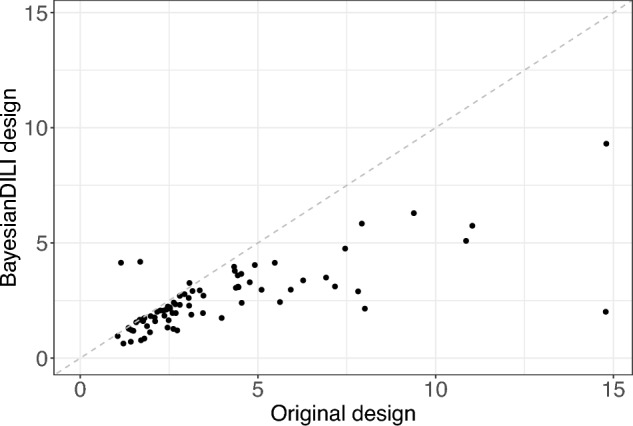
The Scatterplot shows RMSE-values corresponding to the simulation results of 76 substances regarding BayesianDILI and the original used design. Each dot represents one substance. The BayesianDILI design outperforms the original design for the majority of substances

The mean RMSEs of the BayesianDILI design are also smaller than the RMSEs of the original design in the DILI assay (Fig. 10). More precisely, the original design outperforms the BayesianDILI design only for three of 76 substances. These three substances show a rather high toxicity due to their maximal concentration.

Based on the simulation results, the mean distance of the ${\text {EC}}_{50}$-values to the ${\text {EC}}_{50}$-values of its reference curve was calculated for each substance under consideration and design technique. For the sake of comparability between all 76 substances, the ${\text {EC}}_{50}$-values were normalized by the maximal concentration of each substance, respectively. Congenial to the analysis of the RMSE-values, the mean ${\text {EC}}_{50}$-values based on the BayesianDILI design are consistently smaller than the ones based on the original and the log10 design (Suppl. Figure S3 and S4).

### Guideline for the design of cytotoxicity experiments

**Fig. 11:**
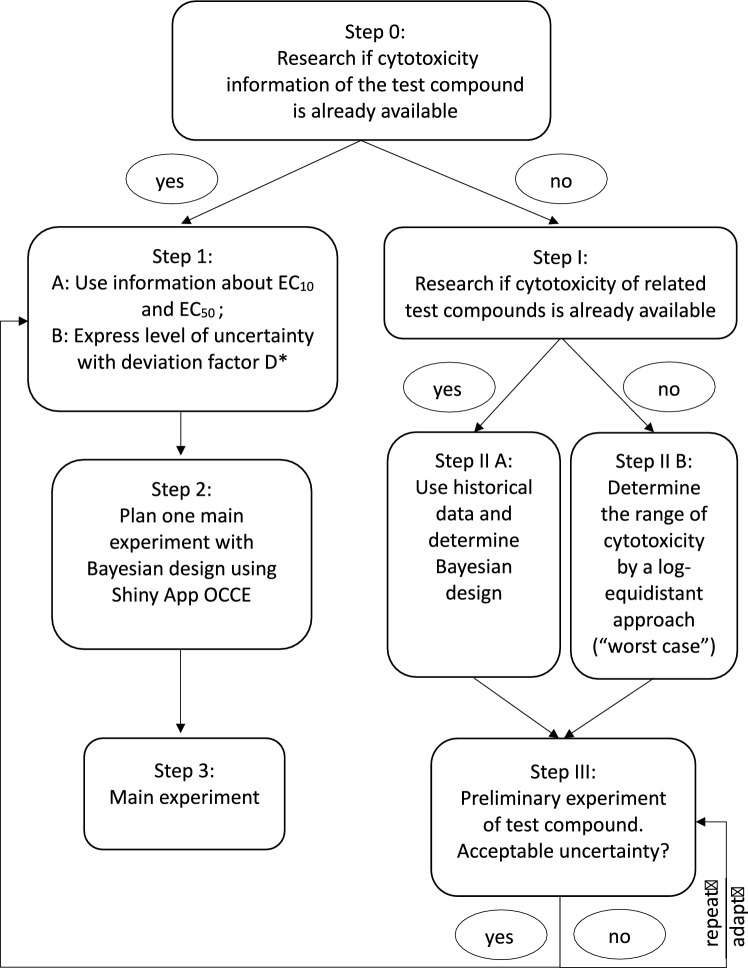
Recommendation for the planning of new cytotoxicity experiments based on the findings of this study. $^*$To ensure a more robust design accounting for uncertainty a deviation factor *D* can be specified. Based on this an upward and downward deviation of the alert concentrations corresponding to exactly this factor can be assumed. If the supplied information of the substance is considered very safe no deviation is assumed, which would result in a deviation factor of 1. The less specific the information is, the higher the factor should be. The deviation factor *D* should not exceed $\frac{x_{\max }}{{\text {EC}}_{50}}$. In the absence of prior knowledge of the test substance a statistician should be consulted for the calculation of Bayesian and adaptive designs

**Fig. 12:**
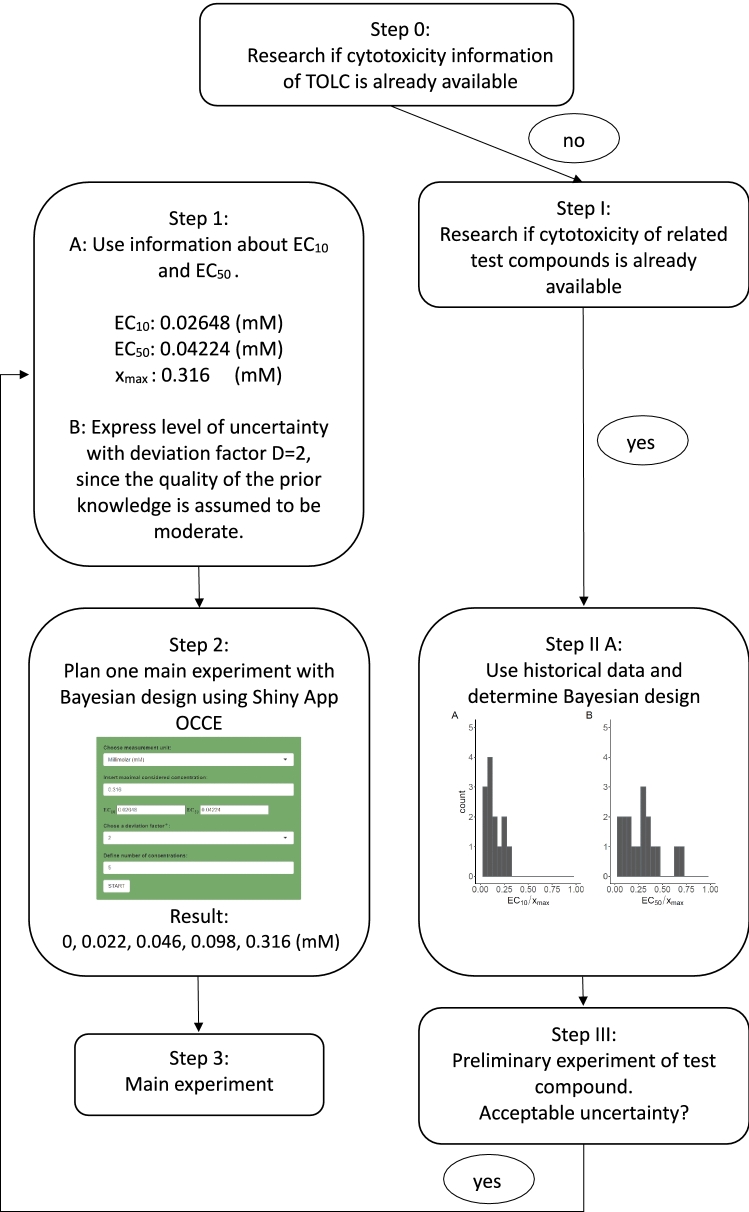
Recommended procedure for the example of tolcapone (TOLC) for the scenario without explicit prior knowledge of the test substance

In cytotoxicity testing, careful consideration must be given to the experimental design, as the concentrations used have a significant impact on the precision of the statistical analysis. In the following, a guideline for the design of cytotoxicity experiments is presented based on our findings (Fig. 11). The recommended procedure for an arbitrary cytotoxicity experiment is presented distinguishing between the scenarios with and without pre-existing direct knowledge about the test substance (Fig. 11). Furthermore, the proceeding based on the guideline is described for the example of the substance tolcapone (Fig. 12).

In the following the individual steps of this guideline will be discussed.

*Step 0: Research if cytotoxicity information of the test compound is already available*. The aim of this initial step is to find out if the specific test compound has already been previously concentration-dependently tested for cytotoxicity. It is not necessary that the test conditions and the tested cell types are identical to the planned experiment. However, the more similar the test conditions are, the better.

If cytotoxicity information about the test substance is known due to previous experiments of the test substance, one proceeds to *Step 1 A: Use information about *${\text {EC}}_{10}$ *and* ${\text {EC}}_{50}$. In this step the alert concentrations can either be identified by reported values or calculated based on previous cytotoxicity data by, e.g. the 4pLL model (Ritz et al. 2015).

*Step 1 B: Express level of uncertainty with deviation factor* *D*. A deviation factor *D* has to be fixed to reflect the possible range of ${\text {EC}}_{10}$- and ${\text {EC}}_{50}$-values. More specifically, all values in $[1/D \cdot {\text {EC}}_{10}, D \cdot {\text {EC}}_{10}]$ and $[1/D \cdot {\text {EC}}_{50}, D \cdot {\text {EC}}_{50}]$ are assumed to be possible values for the ${\text {EC}}_{10}$ and the ${\text {EC}}_{50}$, respectively. Hereby, the deviation factor *D* can be chosen within $[ 1, \frac{x_{\max }}{{\text {EC}}_{50}}]$, in which the highest deviation factor is limited by the maximally considered concentration ($x_{\max }$) and the ${\text {EC}}_{50}$. In this case, it is assumed to be possible that the ${\text {EC}}_{50}$ value and the maximally considered concentration coincide. It should be considered that the deviation factor does not represent an exact natural parameter but to a certain degree includes the subjective assessment of the investigator.

If the information about the substance is precise, a deviation of $D=1$ will be chosen. Setting $D=1$ would represent an unusual situation, in which a) the substance has already been tested with the same cytotoxicity assay and cell line; and b) the ${\text {EC}}_{10}$- and ${\text {EC}}_{50}$-values do not vary between independent experiments (experiments performed on different days). As a consequence a higher deviation factor $D>1$ will be justified in practice. If the previous experiments used the same cytotoxicity test and cell line, the investigator should quantify the deviation factor by the corresponding confidence intervals of independent experiments (for example the basis of the deviation factor will be 2, 3 or 5 if the confidence interval ranges 2, 3 or 5 fold for the individual experiments). This value will be multiplied by an additional factor, e.g., 2 if a different cell line or different experimental conditions (for example a deviating incubation time) were used. The additional factor increases the more the new assay design deviates from the original assay design used to obtain the $\text {EC}$-values. If the quality of previous data justifies a small deviation factor, the shape of the concentration-response curve will be determined more precisely.

In *Step 2: Plan one main experiment with Bayesian design using Shiny App OCCE* the provided Shiny app OCCE (see http://shiny.statistik.tu-dortmund.de:8080/app/occe) can be used. For this purpose ${\text {EC}}_{10}$, ${\text {EC}}_{50}$, the maximally considered concentration $x_{\max }$, deviation factor *D* (from Step 1) and the number of concentrations (*N*) that should be tested will be entered. The result will be a list of *N* concentrations that should be used to conduct the main experiment (Step 3).

In the absence of previous knowledge about the test substance of interest, continue with *Step I: Research if cytotoxicity of related test compounds is already available*. Here related compounds can be substances which belong to a same chemical class and were tested by the similar cytotoxicity test.

*Step II A: Use historical data and determine Bayesian design*. The purpose of this step is to enable a higher density of concentrations around the range where ${\text {EC}}_{10}$- and ${\text {EC}}_{50}$-values can be expected. For the calculation a statistician should be consulted.

*Step II B: Determine the range of cytotoxicity by a log-equidistant approach (“worst case”)*. This “worst case” refers to a situation where almost no information concerning cytotoxicity is available. In this worst case we still know a very wide concentration range, e.g. $10^{-10}$ to 10 M. The cytotoxicity of almost all substances occurs in this range. This means that preliminary experiments have to be performed to narrow down the region of interest. For this purpose an equidistant approach for example with factors of 10 or 100 can represent a useful first step.

*Step III: Preliminary experiment of test compound. Acceptable uncertainty?* In principle, the preliminary experiment can lead to a scenario where a lower concentration will show no toxicity at all while the next higher concentration will show 100% toxicity. In this case the above described deviation factor (*D*) may support the decision whether to directly move to Step 1 or to perform additional preliminary experiments. The concept of the deviation factor can be used to quantify the level of uncertainty by using the range of the confidence intervals of the preliminary experiments. If the deviation factor leads to an exceedance of the maximally considered concentration an additional preliminary experiment should be considered. In general a statistician should be consulted for the calculation of the adaptive designs in this step.

In the following the procedure based on the guideline is described by the example of the substance tolcapone (TOLC) (Fig. 12). In this example it is assumed that no prior knowledge of the substance is available in Step 0. Accordingly, one proceeds with Step I. Since data of 20 related compounds to tolcapone, tested with the same cytotoxicity assay, are available in this example, the corresponding ${\text {EC}}_{10}$- and ${\text {EC}}_{50}$-values are used to construct distributions for possible ${\text {EC}}_{10}$- and ${\text {EC}}_{50}$-values of the test substance TOLC. Based on this a Bayesian design is constructed for the preliminary experiment in Step III. The corresponding uncertainty is assumed to be acceptable since the preliminary experiment showed a confidence interval of the ${\text {EC}}_{50}$ of a nearly 2-fold range. Accordingly, the cytotoxicity information of the preliminary experiment (${\text {EC}}_{10}$, ${\text {EC}}_{50}$, *D*, $x_{\max }$) is used in Step 1. Entering these values in the OCCE app while aiming for five different concentrations results in the calculation of the following five optimal concentrations: 0, 0.022, 0.046, 0.098, 0.316 (mM) (Step 2). The main experiment with at least three technical replicates can then be performed with the corresponding concentrations (Step 3).

### Shiny app OCCE (optimal concentrations for cytotoxicity experiments)

**Fig. 13:**
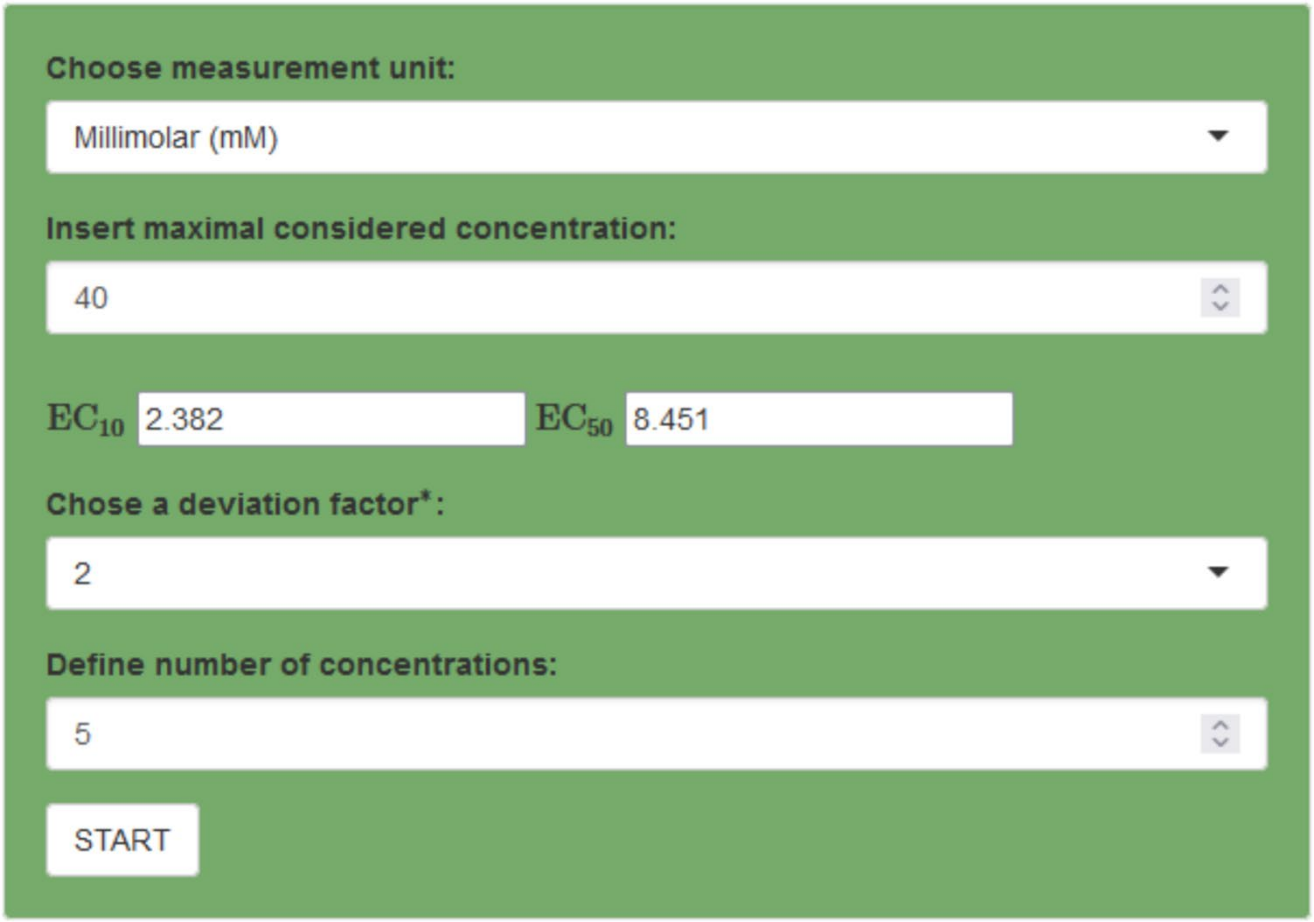
Example of supplied cytotoxicity information in Shiny app OCCE. The resulting optimal concentrations for this example are 0, 2.821, 6.099, 13.503, 40 (mM)

Often the calculation of optimal concentrations for an upcoming cytotoxicity experiment requires deeper knowledge in software programming. To make the calculations easily accessible to everyone, we have developed a Shiny app (see http://shiny.statistik.tu-dortmund.de:8080/app/occe). This Shiny app called **O**ptimal **C**oncentrations for **C**ytotoxicity **E**xperiments (short: OCCE) enables the calculation of optimal concentrations for user-specified situations. First the unit of measurements and the maximally considered concentration for the compound of interest needs to be supplied. Additionally a prior guess of the alert concentrations ${\text {EC}}_{10}$ and ${\text {EC}}_{50}$, as well as a corresponding deviation factor (for details see “Guideline for the design of cytotoxicity experiments”) has to be entered (for an example see Fig. 13). Then the user can specify the desired number of concentrations *N* for the calculation. Afterwards the resulting *N* optimal concentrations based on the supplied knowledge are calculated and returned as a list. Furthermore, details of the parameters and the calculation methodology are given.

**Fig. 14:**
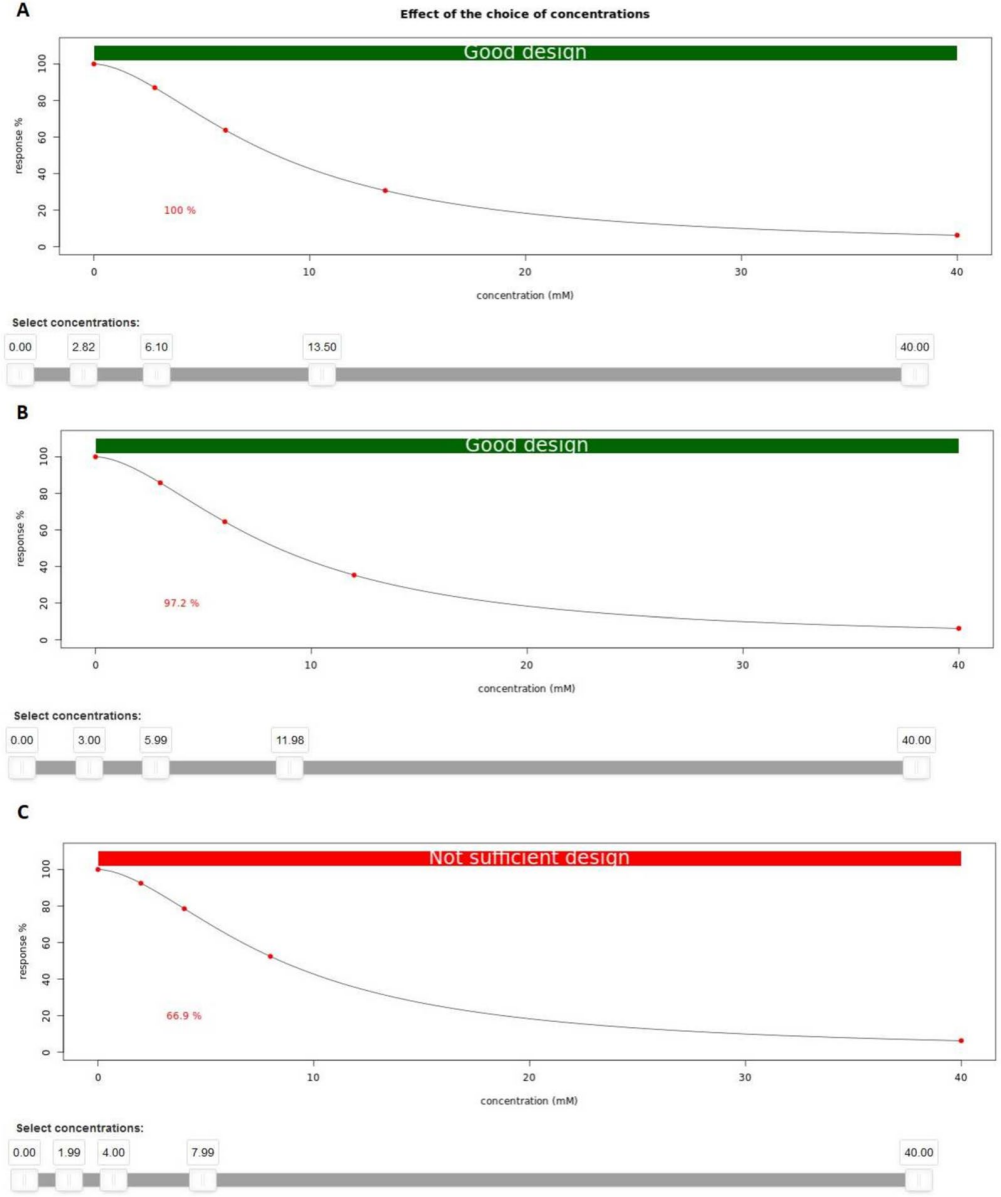
Visualization of the influence of the choice of concentrations based on the corresponding efficiency. **A** Optimal concentrations result in a corresponding efficiency of 100%. **B** A different set of concentrations result in a high efficiency of 97.2%. **C** Different set of concentrations result in a low efficiency of 66.9% and lead to a non sufficient design

Apart from the calculator of optimal concentrations, the OCCE app includes a tool for comparing specific concentrations to the optimal concentrations. In particular, the efficiency of a user-specified design can be evaluated by this app. The higher the efficiency is, the better the chosen design is. This allows for the evaluation of the quality of other designs in comparison to the optimal concentrations. If, for instance, the exact calculated concentration of 2.821 mM is difficult to measure in practice, the user can assess the impact of adjusting the concentration to 3 mM (see Fig. 14). A design with an efficiency over 80% is classified as a good design, while an efficiency of 70 to 80% is sufficient and a design below 70% should not be used to plan a new experiment.

## Discussion and conclusion

This study introduces designs for cytotoxicity experiments for two scenarios: On the one hand, where pre-existing knowledge of the test substance is available and on the other hand, where this is not the case.

For the first scenario, the performance of three different design techniques (log-equidistant, IfADo, and Bayesian) was investigated by the example of valproic acid (VPA). Based on pre-existing knowledge of the ${\text {EC}}_{10}$ and ${\text {EC}}_{50}$, designs with 4–7 concentrations were constructed for each technique, respectively.

To ensure a comparable assessment of the different designs, a reference curve for VPA was determined based on experiments with an unusually high number of 50 different concentrations with 3–6 technical replicates. This reference curve was used to quantify the degree of deviation obtained for the different design techniques in terms of the RMSE and precision of the ${\text {EC}}_{50}$.

The results show that the Bayesian design technique leads to a higher model precision compared to the other techniques for all numbers of concentrations. The Bayesian design leads to RMSEs and ${\text {EC}}_{50}$-values with substantially lower variability and therefore better reproducibility of experimental results. Besides, the log-equidistant design with four to six concentrations leads to high deviations from the reference curve. When incorporating the seventh concentration into the log-equidistant design, it performs considerably better. In contrast to this, no substantial improvement is visible for using more concentrations in the IfADo design. Furthermore, the Bayesian design technique requires by far the smallest sample size for achieving the same precision of the ${\text {EC}}_{50}$ estimate as the other designs.

In addition, the influence of a pre-experiment for the three different design techniques was considered. When the pre-experiment was designed with the Bayesian or log-equidistant design with seven concentrations, no sequential approach consisting of a pre- and a main experiment was necessary, because the gained information was already sufficient in the one-step procedure and not improved by splitting the experiments. For the IfADo design with seven concentrations in the pre-experiment, an improvement using the sequential approach was visible. In general, the sequential approach tends to be superior, if the design of the pre-experiment is suboptimal.

Whereas the pre-existing knowledge in this scenario is based on the ${\text {EC}}_{10}$- and ${\text {EC}}_{50}$-values of previous experiments identified with 0.373, 0.5645, 0.756 and 6, 6.5, 7 mM, respectively, the ${\text {EC}}_{10}$ and ${\text {EC}}_{50}$ observed in the VPA data set are identified with 2.58 and 7.15 mM, so that the previous knowledge deviates from the reference curve in this analysis. Therefore, the designs based on this previous knowledge can be confounded and improved by incorporating adjusted previous knowledge. Nevertheless, the Bayesian technique performed better compared to the other design techniques.

For the second scenario, a large data set consisting of 76 substances (not including VPA) was used to construct a design, called BayesianDILI design, which is based on less specific prior knowledge of these substances. The BayesianDILI design can be adjusted for each test compound in the DILI data set and related compounds by utilizing the corresponding highest considered concentration. The performance of this design was compared to the three former design techniques on VPA data. Here, the BayesianDILI design tailored to VPA, called BayesianDILI_VPA, performed considerably better than the IfADo design, although it did not use specific prior knowledge of the test substance. The BayesianVPA and log-equidistant design showed a higher model precision compared to the BayesianDILI_VPA design, due to their construction on more specific prior knowledge of VPA. In terms of the precision of the ${\text {EC}}_{50}$ the BayesianDILI_VPA design performed worse than the BayesianVPA design but even better than the log-equidistant or IfADo design. It should be considered that the ${\text {EC}}_{50}$ of VPA does not represent the median of the ${\text {EC}}_{50}$-values of all investigated substances, because VPA is comparatively less toxic. The BayesianDILI design could show better results for substances where the ${\text {EC}}_{50}$ is closer to the median.

Considering the performance of the BayesianDILI design for the 76 substances, that were used for its construction, a computer-based simulation study was performed. Hereby, the BayesianDILI design was compared to log-equidistant designs and the original designs used in the DILI data set in terms of RMSE for each substance, respectively. For the vast majority of substances the BayesianDILI design shows a higher model precision than the log-equidistant and originally used design in terms of the RMSE. The analysis of the precision of the ${\text {EC}}_{50}$ showed coherent results. Note that the BayesianDILI design was developed based on prior information of these 76 substances. However, the prior information of a particular substance entered only with weight 1/76 to the prior distribution, so the results also indicate a good performance of the BayesianDILI design for the scenario of no pre-existing knowledge. It should be noted that a Bayesian design could have also been constructed with less prior information. However, the quantity and the quality of the prior knowledge increases the efficiency of the Bayesian design technique.

**Fig. 15:**
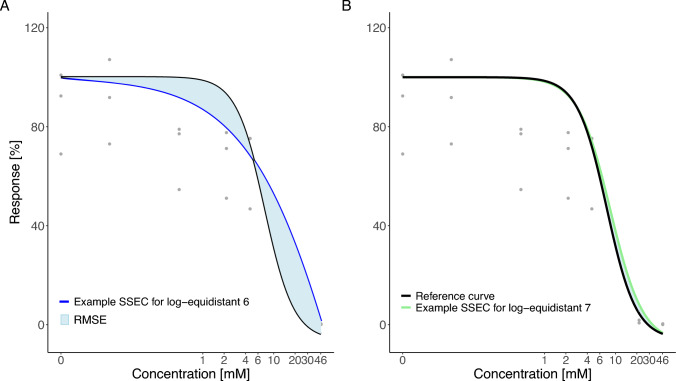
The precision of the log-equidistant design is largely influenced (improved) by inclusion of the specific concentration 21.45 mM. **A** Visualization of the RMSE of an example SSEC for the log-equidistant design with six concentrations compared to the reference curve. **B** Visualization of the RMSE of an example SSEC for the log-equidistant design with seven concentrations compared to the reference curve

Apart from the Bayesian technique, the log-equidistant design with seven concentrations showed a high model precision, at least for VPA. Nevertheless, it has to be noted that this results from incorporating the specific concentration 21.45 mM. In Fig. 15 the improvement of the model precision when incorporating the seventh concentration in the log-equidistant design is shown exemplarily for one SSEC.

In this study, we considered only designs with equal numbers of replicates at each concentration for practical reasons. However, this limits the calculation of optimal designs, as they are not inherently equally weighted by definition.

Furthermore, we prioritized a user-friendly approach by extending the Bayesian design using a simple midpoint interpolation technique. While other methods, such as optimizing additional concentrations based on the criterion value, could have been applied, they would likely yield similar concentrations. Our approach ensures that the original Bayesian design concentrations are preserved while broadly covering the design space. This is advantageous in practice, as it facilitates the identification of the entire concentration-response curve.

The results clearly demonstrate the advantages of using techniques of optimal design theory in practice. The usage of the Bayesian technique with and without incorporating pre-existing knowledge of a specific test substance resulted in the most precise statistical inference of the corresponding experiments. Here, the approach is used for mostly hepatotoxic substances, but it can be easily adjusted to different substance sets.

To provide an easy calculation tool, we developed a Shiny app that allows users to easily determine optimal concentrations for future cytotoxicity experiments. Additionally, this Shiny app enables the comparison of various concentrations against the optimal values.

## Supplementary Information

Below is the link to the electronic supplementary material.

Supplement Sequential analysis (pdf 96 KB)

Supplement Figures (pdf 229 KB)

Supplement Data (xlsx 140 KB)

## Data Availability

The data set as it is used in this publication can be found in the supplementary material (see Supplement Data).

## Declarations

### Code availability

The corresponding Shiny app can be found online openly accessible at http://shiny.statistik.tu-dortmund.de:8080/app/occe.
