# Supplementary material for: Design of optimal concentrations for in vitro cytotoxicity experiments
Source: Arch Toxicol. 2024 Nov 16;99(1):357–76. doi: 10.1007/s00204-024-03893-1 (PMC11748471; doi:10.1007/s00204-024-03893-1)
Supplement: Supplementary file 1 — Supplement Sequential analysis (pdf 96 KB) [file 204_2024_3893_MOESM1_ESM.pdf]

Supplementary material

Sequential approach improves IfADo design

Concentration-dependent toxicity testing might be improved by using a sequential procedure, in which the main experiment is done after the conduction of a pre-experiment. The pre-experiment is used to obtain knowledge about the EC_{10}-value and the EC_{50}-value of the test substance. Based on this information, the main experiment is performed to gain detailed knowledge about the cytotoxicity of the test substance.

To analyze the influence of the choice of the design conditions the design is varied in the pre-experiment with three possibilities (log-equidistant, IfADo and Bayesian) with seven concentrations each. Based on the VPA data set scenario specific estimated curves (SSECs) as described in Section Results were calculated 3000 times with three observations at each concentration. Based on each of those SSECs the confidence intervals of the corresponding EC_{10} and EC_{50} are calculated. With the help of this, parameter combinations were constructed to incorporate uncertainty and a prior distribution is established based on the parameter combinations. Afterwards a Bayesian D-optimal design with seven concentrations can be developed based on this prior distribution (which is unique for each SSEC) assuming equal weights. To make sure the resulting concentrations are available in the VPA data set, the concentrations of the SSEC specific Bayesian design were fixed to the seven different nearest available concentrations of the data set. The main experiment then is conducted with these specific concentrations resulting from the Bayesian technique. In a last step, three observations per concentration were drawn randomly from all data points available in the data set to imitate a main experiment. Additionally, a 4pLL-model is fitted to the new data points. This model then can be compared to the reference curve via RMSE and precision of the EC_{50}. Note that the other design techniques were not used for the design of the main experiment, as they performed worse than the Bayesian design technique with seven concentrations in the previous analyses.

The RMSEs and EC_{50}-value of the sequential approach were compared to the RMSEs and the EC_{50}-value of the non-sequential procedure, in which a main experiment with six replicates at each concentration was conducted based on either the Bayesian, log-equidistant or IfADo design with seven concentrations. Note that for the sake of comparability, the number of replicates was chosen such that both the non-sequential and the sequential procedures result in the same total sample size.

The performance of the log-equidistant design and the IfADo design is improved by the sequential approach, whereas the performance of the Bayesian design is not affected (see Fig. 1). More precisely, the results show that neither the median RMSE nor the median EC_{50}-value is improved by the sequential approach if the pre-experiment had already been based on the Bayesian technique. In contrast, the RMSEs and the EC_{50}-values of the IfADo design were improved substantially by the sequential procedure compared to the non-

sequential approach. However, the sequential IfADo approach remains worse than the non-sequential Bayesian approach. Focusing on the log-equidistant design, the sequential approach reduces the number and extension of outliers in terms of RMSEs and EC_{50}-values.

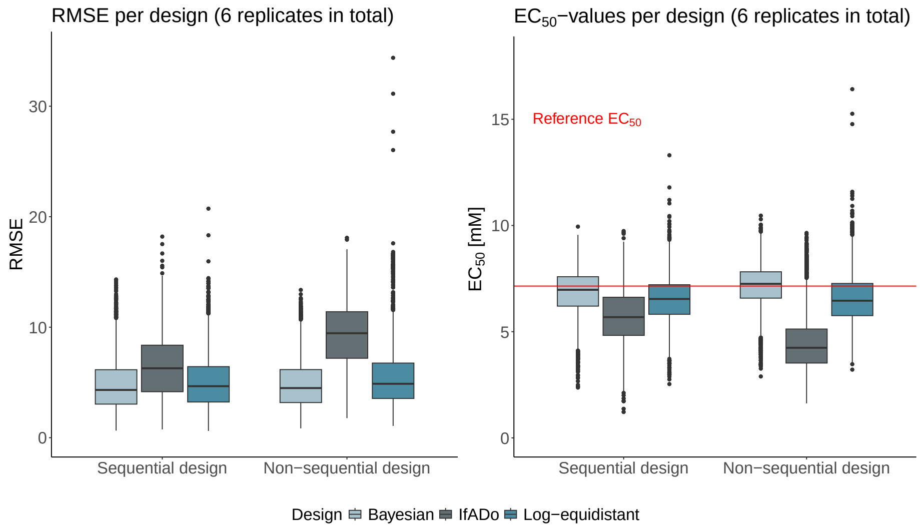

Figure 1: IfADo and log-equidistant design are improved by the sequential design procedure in terms of precision and reduction of outliers.
A: RMSE-values for sequential and non-sequential approach grouped by pre-experimental condition (different designs), B: EC_{50}-values for sequential and non-sequential approach grouped by pre-experimental condition (different designs). The red line corresponds to the reference EC_{50}.
