# Supplementary material for: Design of optimal concentrations for in vitro cytotoxicity experiments
Source: Arch Toxicol. 2024 Nov 16;99(1):357–76. doi: 10.1007/s00204-024-03893-1 (PMC11748471; doi:10.1007/s00204-024-03893-1)
Supplement: Supplementary file 2 — Supplement Figures (pdf 229 KB) [file 204_2024_3893_MOESM2_ESM.pdf]

Supplementary material

Figures

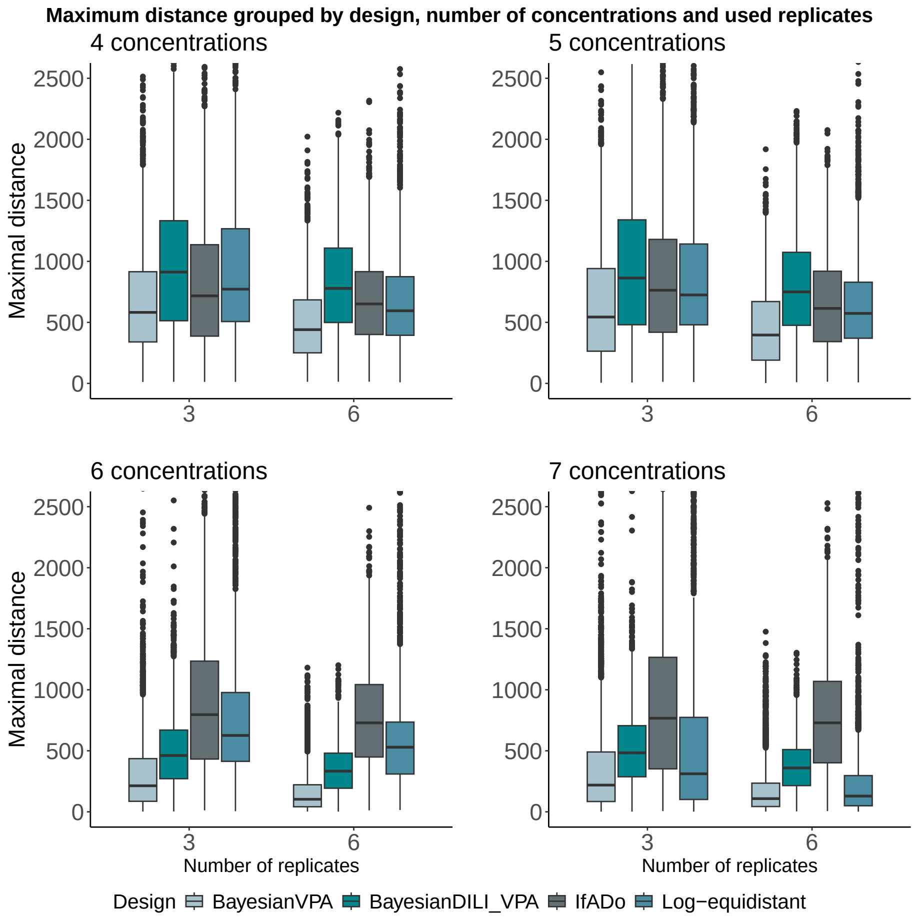

Figure S1: Maximum distance grouped by design, number of concentrations, and the use of replicates. This figure presents only an extract of the data, excluding extreme outliers across all designs.

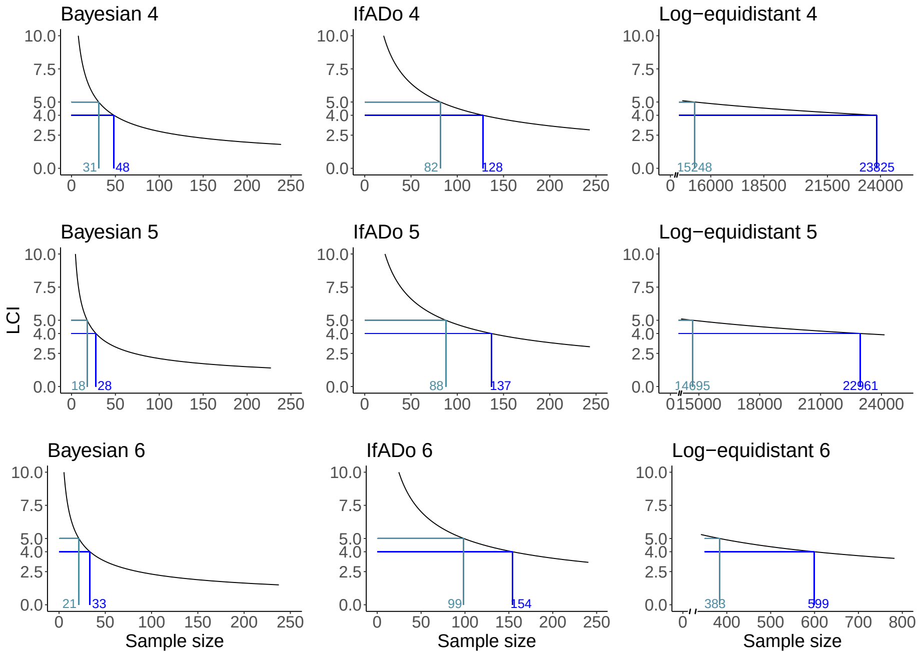

Figure S2: The relation between the sample size and the length of the confidence interval (LCI) of the EC_{50}-value is shown grouped by design and number of concentrations. The blue lines indicate the number of observations required to achieve a specific level of precision for the EC_{50} (LCI). To achieve the same precision of the EC_{50} the Bayesian design requires considerably less observations than the other two design techniques.

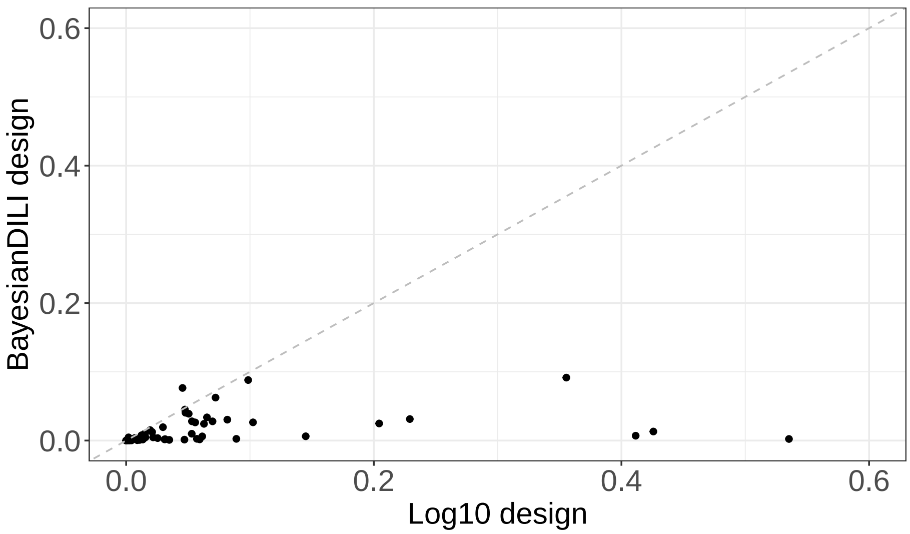

Figure S3: An extract of the simulation results from all 76 test compounds is shown regarding the precision of the EC_{50} for the BayesianDILI compared to the log10 design. The precision is measured by the mean distance of the simulated EC_{50}-values compared to the reference EC_{50} for each substance. Each dot represents one substance. For the sake of comparability, too extreme values are not shown in this plot. The BayesianDILI design outperforms the log10 design for the majority of substances.

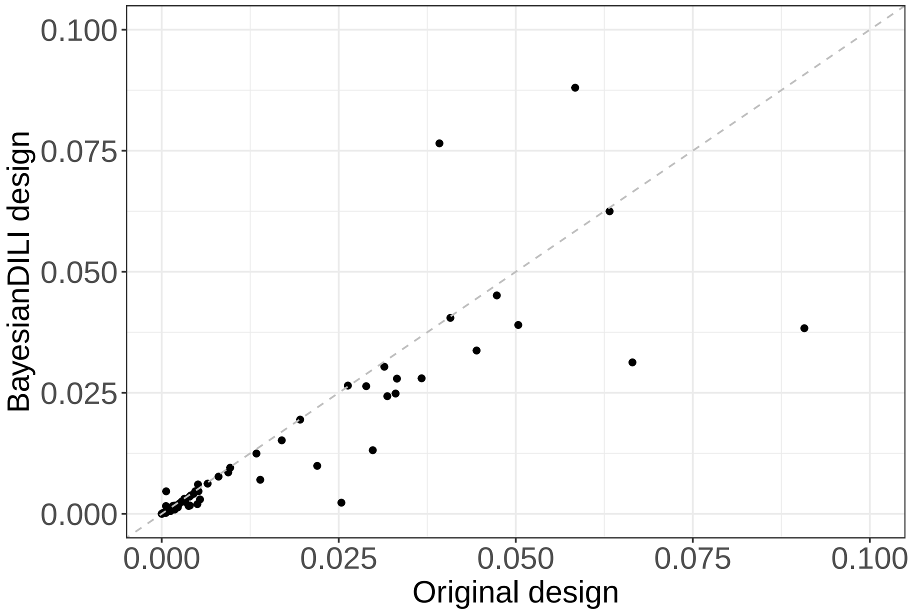

Figure S4: An extract of the simulation results from all 76 test compounds is shown regarding the precision of the EC_{50} for the BayesianDILI compared to the original used design. The precision is measured by the mean distance of the simulated EC_{50}-values compared to the reference EC_{50} for each substance. Each dot represents one substance. For the sake of comparability, too extreme values are not shown in this plot. The BayesianDILI design outperforms the original design for the majority of substances.
